# Altered cortical thickness of the superior frontal gyrus and fusiform gyrus in individuals with subthreshold social anxiety

**DOI:** 10.1038/s41598-023-49288-7

**Published:** 2023-12-09

**Authors:** Byoung-Ho Kim, So-Young Park, Chun Il Park, Minji Bang, Hyun-Ju Kim, Sang-Hyuk Lee

**Affiliations:** 1 Department of Psychiatry, CHA Bundang Medical Center, CHA University School of Medicine, 59 Yatap-Ro, Bundang-Gu, Seongnam-Si, Gyeonggi-Do 463-712 Republic of Korea; 2 CHA University School of Medicine, Seongnam, Republic of Korea

**Keywords:** Psychology, Social neuroscience

## Abstract

Subthreshold social anxiety (SSA) is a condition in which individuals experience social anxiety that does not reach the threshold required for a clinical diagnosis of a social anxiety disorder (SAD). Although SSA may not impair lives as severely as SAD, it can affect social functioning. However, only a few studies focused on structural neural correlates of SSA. We recruited 65 individuals with SSA and used the Leibowitz Social Anxiety Scale to assess their social and performance anxiety levels and other relevant measures of social anxiety. Voxel-wise whole-brain correlational analyses showed a positive association between the cortical thickness (CT) of the superior frontal gyrus (SFG) and social anxiety levels and a negative correlation between the CT of the fusiform gyrus (FG) and performance anxiety levels in individuals with SSA. Exploratory Pearson’s correlation analyses showed significant positive correlations between the CT of the SFG and Generalized Anxiety Disorder-7 total scores and negative associations between the CT of the FG and Beck Anxiety Inventory total scores. Our study provides insight into the neural basis of SSA, particularly performance anxiety, by highlighting the association between CT in specific brain regions and SSA characteristics.

## Introduction

Social anxiety, or intense fear and avoidance of social or performance situations, is related to distress, which ranges from low or moderate nervousness to severe and incapacitating dread^1,2^. People with extreme levels of social anxiety and persistent avoidant behaviors for at least six months may meet the diagnostic criteria for social anxiety disorder (SAD)^1^. Social anxiety symptoms typically lead to substantial distress or impairment in various areas of life, such as social activities, work, school, or relationships^3–5^.

Performance anxiety is the fear of speaking or performing in public among individuals who must speak or perform in front of an audience. It is also regarded as a subtype (performance-only subtype) of social anxiety in the *Diagnostic and Statistical Manual of Mental Disorders, Fifth Edition* (*DSM-5*)^4^. It is prevalent in various fields, including the performing arts, sports, and public speaking^6,7^. People with high levels of performance anxiety tend to have high expectations of themselves and desire to perform flawlessly without any mistakes^8^. Fear rooted in concern about making mistakes and being negatively evaluated by others can be intense^9,10^. The fear of negative evaluation and pressure to perform perfectly can impact a person’s self-esteem and confidence considerably, which can have detrimental effects on their professional career^10,11^.

Subthreshold social anxiety (SSA) is a condition in which individuals experience social anxiety similar to that seen in SAD, but the symptoms do not reach the threshold required for a clinical diagnosis of SAD^12^. A previous study reported that the prevalence of SSA was higher than that of SAD^13^. The one-year prevalence of SSA was 3.0%, while SAD's was 2.0%^13^. SSA might impair both interpersonal relationships and social life, although to a somewhat lesser extent than SAD and could lead to the progression of SAD^14,15^. Despite the potential seriousness of its effects, the neurobiological mechanisms underlying SSA remain unclear. Furthermore, a few studies have examined the relationship between SSA-related brain regions and other psychological characteristics, including excessive worry or anxiety.

In recent years, the construct of social anxiety has existed on a continuum rather than as a discrete, dichotomous concept. This shift aligns with the Research Domain Criteria (RDoC) framework proposed by the National Institute of Mental Health^16,17^. Factor analysis findings suggest that the latent structure of social anxiety appears to be similar for people with and without SAD^18^. Neuropsychologically, attentional biases related to social anxiety at clinical levels of severity have also been found to play a role at non-clinical levels of severity^19,20^. This overlap may implicate the continuity in the underlying neural mechanisms related to social anxiety across the spectrum of severity^20,21^.

Meanwhile, functional magnetic resonance imaging (MRI) studies have revealed that the amygdala, insular cortex, and hippocampus play a substantial role in social anxiety^22–25^. A meta-analysis of structural MRI found larger gray matter volume (GMV) in the cortical regions, such as the superior frontal gyrus (SFG) and fusiform gyrus (FG), and smaller GMV in the subcortical regions, including the putamen and thalamus, of patients with SAD^26^. Furthermore, voxel-wise analyses revealed increased cortical thickness (CT) in the frontoparietal cortex of patients with SAD^27^. Patients with SAD show increased cortical surface area (SA) in the prefrontal cortex, lateral orbitofrontal cortex, and superior temporal gyrus^28^. Interestingly, a previous study demonstrated that dissociated morphological alterations were prominent in patients with SAD, as demonstrated by decreased CT and increased SA in the prefrontal cortex^28^.

However, in individuals with SSA, only a few studies have focused on structural brain alterations^29,30^. One study found significant positive correlations between social anxiety levels and the amygdala and striatal volume in a small sample of healthy female individuals^29^. Another study also showed that CT or SA in the FG was significant and negatively related to social anxiety levels measured via a self-report questionnaire, demonstrating a lower number of brain regions with decreased CT among individuals with SSA when compared to those in patients with SAD^30^. From a clinical perspective, as there are individuals with SSA, who do not reach the diagnosis of SAD, but suffer from social anxiety symptoms along a continuum of social anxiety dimensions, it may be crucial to alleviate their discomfort and to understand the pathophysiology of SSA.

Therefore, this study aimed to identify the structural differences in the brain based on the degree of social anxiety (or performance anxiety), particularly within individuals with SSA. Because the findings of previous studies on the association between social anxiety (or performance anxiety) and structural alterations are discrepant and inconsistent concerning individuals with SSA, we conducted voxel-wise whole-brain analyses instead of a regions of interest study. Furthermore, we investigated whether the dissociations between CT and SA in patients with SAD are also prominent in individuals with SSA. We hypothesized that (1) individuals with SSA (or performance anxiety) would show altered GMV, CT, and SA (i.e., a lower number of regions compared to SAD) in the above-mentioned regions, which are known to be related to social anxiety; (2) dissociated morphological alterations between CT and SA would be present in the prefrontal regions of individuals with SSA; and (3) there would be significant correlations between the brain regions implicated in SSA (or performance anxiety) and psychological measures of individuals with SSA.

## Results

### Sociodemographics and clinical characteristics

Table 1 summarizes the participants’ sociodemographic and clinical characteristics**.** Among the 65 participants (37 women and 28 men), most reported having a bachelor’s degree or higher. The social anxiety level (mean LSAS score) was 21.35, and the performance anxiety level (performance anxiety subscale mean scores) was 12.36 in individuals with SSA.

**Table 1:** Sociodemographics and clinical characteristics of individuals with subthreshold social anxiety (*N* = 65).

Demographics	Mean (± SD) or count (%)
Age at scan (years)	36.05 (± 9.16)
Gender (male/female)	28 (43.10) / 37 (56.90)
Years of education (years)	17.12 (± 2.27)
Intracranial volume (cm)	1536.02 (± 124.99)

Clinical characteristics	Mean (± SD)
Levels of social anxiety	
LSAS total scores	21.35 (± 11.75)
1. LSAS-fear	9.54 (± 5.50)
2. LSAS-avoidance	10.20 (± 5.21)
Levels of performance anxiety^a^	12.36 (± 10.18)
Excessive worry (GAD-7 total scores)	9.35 (± 2.72)
Anxiety (BAI total scores)	4.36 (± 5.08)
Depression (BDI-II total scores)	6.17 (± 5.69)

SD, standard deviation; LSAS, Leibowitz Social Anxiety Scale; GAD-7, Generalized Anxiety Disorder-7; BAI, Beck Anxiety Inventory; BDI-II, Beck Depression Inventory-II.

^a^The value of performance anxiety is the sum of 13 LSAS items related to performance anxiety.

### Pearson’s correlation analyses among clinical symptom measurements

Table 2 shows Pearson’s correlation analysis results among clinical symptom measurements, including social anxiety (and performance anxiety) levels, Generalized Anxiety Disorder-7 (GAD-7), Beck Anxiety Inventory (BAI), and Beck Depression Inventory (BDI-II) in individuals with SSA**.** There are significant positive correlations between social anxiety (and performance anxiety) levels and symptom severity levels in other clinical measurements (e.g., GAD-7, BAI, or BDI-II).

**Table 2:** Pearson’s correlation analyses among the clinical measurements of individuals with subthreshold social anxiety.

	1	2	3	4	5
1. Levels of social anxiety (LSAS total scores)	–	–	–	–	–
2. Levels of performance anxiety	0.952**	–	–	–	–
3. Excessive worry (GAD–7 total scores)	0.527**	0.492**	–	–	–
4. Anxiety (BAI total scores)	0.495**	0.446**	0.623**	–	–
5. Depression (BDI-II total scores)	0.419**	0.389**	0.729**	0.643**	–

LSAS, Leibowitz Social Anxiety Scale, GAD-7, Generalized Anxiety Disorder-7; BAI, Beck Anxiety Inventory; BDI-II, Beck Depression Inventory-II.

**p < 0.01.

### Voxel-wise correlational analyses between the social anxiety (or performance anxiety) levels and the brain structures in individuals with SSA

**Figure 1:**
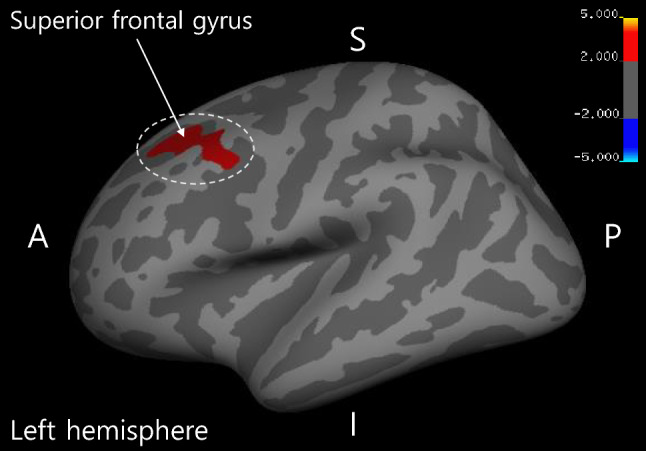
Result of voxel-wise analysis for social anxiety levels. The CT of the SFG is significantly positively correlated with the LSAS total scores in individuals with SSA. *Note* MNI coordinate *X* = -23.8, *Y* = 16.5*, Z* = 39.1. Monte Carlo simulations correction (CWP < 0.05). The color bar shows a logarithmic scale of *p* values (− log10). CT, cortical thickness; SFG, superior frontal gyrus; LSAS, Leibowitz Social Anxiety Scale; SSA, subthreshold social anxiety; MNI, Montreal Neurological Institute, CWP: cluster-wise probability.

We performed voxel-wise correlation analyses between social anxiety scores and structural brain measures, including the brain regions’ GMV, CT, and SA. A significant positive correlation was observed between the Liebowitz Social Anxiety Scale (LSAS) total scores and the CT of the SFG in individuals with SSA [cluster-wise probability (CWP) = 0.0026]. This association remained significant after adjusting for age at the time of the MRI scan, gender, and intracranial volume (ICV) (CWP = 0.0056; Fig. 1). However, both SA and GMV were not significantly correlated.

**Figure 2:**
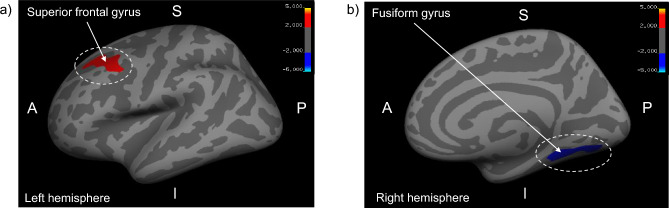
Result of voxel-wise analysis for performance anxiety levels. (**a**) The CT of the SFG is significantly positively correlated with performance anxiety levels in individuals with SSA (MNI coordinate *X* =—23.4, *Y* = 17.8, *Z* = 38.3; Monte Carlo simulations correction, CWP < 0.05). (**b**) The CT of the FG is significantly negatively correlated with the LSAS performance anxiety scores in individuals with SSA (MNI coordinate *X* = 34.4, *Y* = -48.9, *Z* = -11.4; Monte Carlo simulations correction, CWP < 0.05). *Note*: The color bar shows a logarithmic scale of *p* values (− log10). CT, cortical thickness; SFG, superior frontal gyrus; SSA, subthreshold social anxiety; MNI, Montreal Neurological Institute; CWP, cluster-wise probability; FG, fusiform gyrus; LSAS, Leibowitz Social Anxiety Scale.

The LSAS performance anxiety scores showed significant correlations with the CT of several brain regions in individuals with SSA (Fig. 2). They were significantly and positively correlated with the CT of the SFG (CWP = 0.0006), even after controlling for age, gender, and ICV (CWP = 0.0024). Furthermore, the LSAS performance anxiety scores were significantly negatively correlated with the CT of the FG (CWP = 0.0006), even after controlling for age, gender, and ICV (CWP = 0.0046). No significant correlations were found with other structural brain measures, including GMV and SA.

### Exploratory Pearson’s correlation analyses between the cortical thickness of the significant brain regions and social anxiety-relevant psychological scores in individuals with SSA

**Figure 3:**
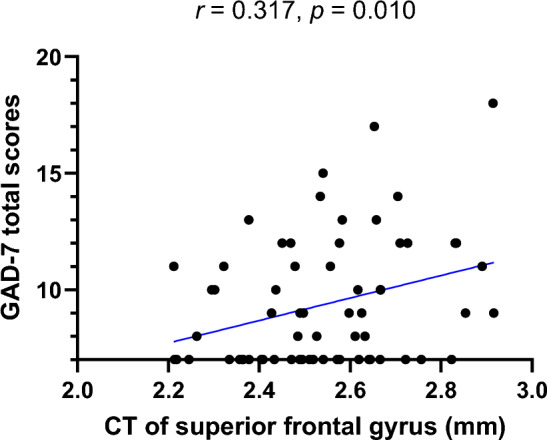
Exploratory Pearson’s correlation analyses showed significant positive associations between the CT of the SFG related to social anxiety levels and the GAD-7 total scores in the individuals with SSA. CT, cortical thickness; SFG, superior frontal gyrus; GAD-7, Generalized Anxiety Disorder-7; SSA, subthreshold social anxiety.

We performed Pearson’s correlation analyses between the CT of significant brain regions related to levels of social anxiety (or performance anxiety) and the total scores regarding the GAD-7, BAI, and BDI-II for individuals with SSA (Supplementary Table 1). Figure 3 shows significant positive correlations between the CT of the SFG and the total scores of the GAD-7 (*r* = 0.317, *p* = 0.010). Additionally, the significance was maintained after Spearman’s correlation analyses between the CT of significant brain regions and the total GAD-7 scores in individuals with SSA.

**Figure 4:**
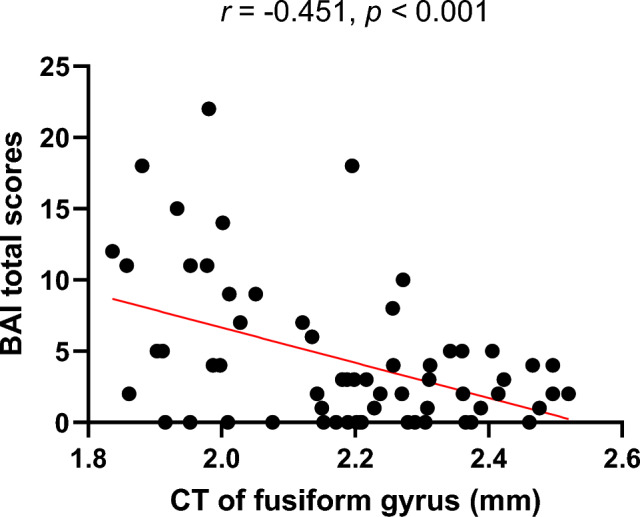
Exploratory Pearson’s correlation analyses showed significant negative associations between the CT of the FG related to performance anxiety levels and the BAI total scores in individuals with SSA. CT, cortical thickness; FG, fusiform gyrus; BAI, Beck Anxiety Inventory; SSA, subthreshold social anxiety.

The thinner the CT of the FG, the higher the total BAI score in individuals with SSA (*r* = -0.451, *p* < 0.001; Fig. 4). Additionally, the significance was maintained after Spearman’s correlation analyses between the CT of significant brain regions and the total BAI scores in individuals with SSA. The CT of the FG did not show significant correlations with other clinical variables.

## Discussion

Our study investigated structural brain alterations, such as the CT of the SFG and FG, and their relations with the clinical characteristics of individuals with SSA. We found significant positive correlations between the CT of the SFG and excessive worry levels. The thinner the CT of the FG, the higher the anxiety level in individuals with SSA. The CT of the SFG and FG may thus be associated with social anxiety and anxiety-related behaviors in individuals with SSA.

This study suggests that individuals with SSA exhibit positive associations between social anxiety and the CT of the SFG, which is located in the anterior portion of the dorsomedial prefrontal cortex. This brain region is involved in cognitive functions such as working memory and problem-solving^31–33^. Although little research has directly linked cognitive functions to social anxiety, structural MRI research has shown a negative correlation between social anxiety levels and CT in the SFG^28^. Interestingly, the increased CT in the dorsolateral prefrontal and parietal cortices of patients with SAD could be a compensatory response to coping efforts in emotion regulation^27^. Similarly, the increased CT observed in the SFG of individuals with SSA in the current study may be interpreted as a compensatory response to the challenges posed by social anxiety. However, we did not observe significant changes in the SA of the SFG among individuals with SSA compared to a previous SAD neuroimaging study^28^ showing increased CT and decreased SA in the SFG region. This suggests that the structural alterations of the SA in the SFG in SSA may differ from those in SAD.

Our study showed significant positive correlations between the CT of the SFG and excessive worry levels in individuals with SSA. Excessive worry is a psychological trait associated with dysfunction of the prefrontal cortex, including the SFG^34^. The positive correlations between the CT of the SFG and excessive worry scores suggest that alterations in SFG morphology may contribute to the cognitive and emotional processes underlying excessive worry.

Meanwhile, the negative correlation between the CT of the FG and performance anxiety levels in individuals with SSA suggests that those with elevated performance anxiety may exhibit thinner cortical tissue in this region. The FG is a key region involved in processing emotional faces and is considered a central node within the brain’s facial processing circuits^35,36^. Previous research has shown significant negative correlations between the CT or SA of the FG and social anxiety levels in patients with SAD^30^. This aligns with prior research suggesting a link between FG thinning and elevated levels of performance anxiety, potentially reflecting difficulties in processing social cues and emotional facial expressions^37^. This thinning in the FG may indicate aberrant social information processing, specifically related to facial expressions.

Our study revealed that a thinner CT in the FG correlated with higher anxiety levels in individuals with SSA. The FG is involved in processing emotional facial information and receiving direct input from the amygdala^38^. Recent research has demonstrated a strong relationship between the amygdala and visual cortical activation, such as facial expressions, during emotional stimulant processing^39^. Our findings confirm that thinning of the FG is associated with abnormal social information processing, particularly in individuals with SAD^40^. Therefore, cortical thinning of the FG influences anxiety regulation by affecting the amygdala in individuals with SSA. Structural differences in the FG may affect the processing and regulation of emotional stimuli, potentially leading to dysregulated emotional responses and heightened anxiety levels in social situations.

As mentioned previously, the brain regions associated with SAD are the amygdala, hippocampus, insular cortex, SFG, FG, putamen, thalamus, and frontoparietal cortex. However, our study found the lower numbers of the brain regions (e.g., two regions: SFG and FG) correlated with social anxiety in individuals with SSA when compared to those with SAD. The reason is unclear; however, we can assume that the severity of social anxiety may have played a role in the observed differences. This heightened severity could have led to more pronounced structural alterations in brain regions associated with social anxiety. Secondly, differences in the study design, such as analysis methods and sample sizes between our study and those focused on SAD, may have contributed to the differences in results.

Our findings showed the following clinical significance: even if individuals have SSA, they show morphological and structural alterations in the SFG or FG that are relevant to their psychological distress. These changes may lead to significant functional impairments in social relationships and related changes in cognitive and behavioral manifestations^41^. Therefore, consideration of individuals seeking SSA treatment is needed, even if they are not diagnosed with SAD.

This study has several limitations. First, the sample size was relatively small and limited to SSA without comparison groups—such as among patients with SAD and healthy controls without SSA. Therefore, further studies should investigate the association between social anxiety levels and structural brain alterations in larger and more diverse groups. Second, because a self-report questionnaire was used to assess psychological characteristics, a neuroimaging study using clinician-administered measures will be needed in the future. Nevertheless, all the assessments in our study were valid and objective indicators that had been repeatedly verified. Third, because we used a correlation analysis to determine the neural correlates of SSA, the neural correlates do not necessarily indicate the cause of SSA. In addition, as there are changes in the autonomic nervous, immune, and endocrine systems as well as in the brain, it was difficult to prove causality by controlling for these changes, which may be involved in SSA. Fourth, our study showed that the correlation between levels of social anxiety and performance anxiety is statistically significant. This may suggest that it is difficult to statistically separate the constructs of social anxiety severity and performance anxiety severity. Therefore, the study results need to be interpreted carefully, and well-designed integrative research is required in the future.

In conclusion, heightened levels of social anxiety, including performance anxiety, in individuals with SSA are associated with specific structural changes in the brain—specifically, cortical thickening in the SFG and thinning in the FG. Cortical thickening in the SFG may be a compensatory response to manage social anxiety, while the observed thinning of the FG may indicate difficulties with facial-emotional processing among individuals with SSA.

## Materials and methods

### Participants

We recruited 65 right-handed Korean individuals aged between 19 and 54 years from the Department of Psychiatry at the CHA Bundang Medical Center of CHA University (Gyenoggi-do, Republic of Korea) through advertisements between October 2014 and November 2021. Participants were excluded if they met any of the following criteria: (1) history of head trauma; (2) neurological disorders; (3) major medical illness; (4) current or past substance abuse and dependence; (5) current or past other major psychiatric disorders (e.g., schizophrenia spectrum disorders, major depressive disorders, or bipolar disorders); (6) current or past anxiety disorders, including SAD, panic disorder, agoraphobia, generalized anxiety disorder, and specific phobia; (7) ongoing psychological treatment or recently started pharmacological treatment; (8) MRI contraindications (e.g., metallic implants, claustrophobia); and (9) pregnancy. We used the Structured Clinical Interview for the *DSM-5* to assess the presence of anxiety disorders and other psychiatric conditions, ensuring that only individuals without these disorders were included in the study^42,43^.

All study procedures were approved and reviewed by the Institutional Review Board of the CHA Bundang Medical Center (IRB no. 2019-05-030). Participants were provided with a detailed explanation of the study, and their written informed consent was obtained. The study was conducted in accordance with the Declaration of Helsinki. Furthermore, the principles of Good Clinical Practice were followed to ensure the study’s integrity, reliability, and ethical conduct.

### Social and performance anxiety assessments

The LSAS is a well-validated assessment tool for measuring symptom severity of social and performance anxiety by assessing fear levels and avoidance of social situations^44^. The clinical utility of the LSAS has been supported for use with individuals with SSA^41,45^. One study demonstrated the effectiveness of the LSAS in detecting changes in SSA levels among a sample of college students^45^. In addition, the LSAS was useful in identifying alterations in functional connectivity in individuals with SSA in a neuroimaging study^41^. The questionnaire consists of 24 items assessing fear and avoidance in different social situations on a 4-point scale (ranging from 0 = *none or never* to 4 = *severe fear or usually*, 68–100%)^44^. This scale can be divided into two subscales: 11 items for social interaction anxiety and 13 items for public performance anxiety. Our study included only clinical participants who complained of SSA levels but who were not diagnosed with SAD through experienced psychiatrist interviews. Their total LSAS score was below 68, which was consistent with previous research findings. To assess the levels of social and performance anxiety, all participants completed the LSAS. The Cronbach’s *α* coefficients for the LSAS total score were high (0.94), and those for the subscales ranged from 0.71 to 0.91^46^.

### Other clinical characteristics assessments

To investigate other clinical characteristics, we used the GAD-7^47^, BAI^48^, and BDI-II^49^. The GAD-7 is a self-report questionnaire designed to assess an individual’s level of excessive worry in the previous two weeks^50^, consisting of seven questions. The Korean versions of the BAI and BDI-II were used to evaluate psychological distress, including depression and anxiety. The BAI has 21 self-reported inventories for assessing the severity of clinical anxiety on a scale of 0–3^48^. Similar to the BAI, the BDI-II consists of 21 self-administered items ranging from 0 to 63,^49^. The total BAI and BDI-II scores reflect the severity of anxiety and depression.

### Neuroimaging data acquisition and analyses

The GMV, CT, and SA of all participants’ whole brains were analyzed by T1-weighted MRI using FreeSurfer’s (v7.4.1; https://surfer.nmr.mgh.harvard.edu/) surface-based pipeline. All study participants underwent brain MRI through a 3.0 Tesla GE Signa HDxt scanner (GE Healthcare, Milwaukee, WI, USA). High-resolution structural images were acquired using a three-dimensional, T1-weighted, fast-spoiled, gradient-recalled echo sequence (repetition time = 6.3 ms; echo time = 2.1 ms; flip angle, 12°; field of view, 256 mm; matrix, 256 × 256; and voxel size, 1 × 1 × 1 mm^3^). Appropriate registration, cortical parcellation, and subcortical segmentation were performed during the data preprocessing. The cortex was automatically parcellated into 34 gyral regions per hemisphere using FreeSurfer. The Desikan-Killiany atlas was used for this parcellation^51^. The results were visually inspected for quality control, and no manual editing was required.

After a quality check, cortical and subcortical volumes were measured for each individual, along with the total GMV and total ICV. In addition, the CT for each surface vertex was computed as the shortest distance between the white matter and the pial surface. The cortical maps were then smoothed using a Gaussian kernel with a full width at half maximum of 10 mm. The SA, which represents the area of the vertex on the gray matter surface, was measured at the pial level. It was calculated as the mean of the areas of the tessellated triangles touching that vertex.

### Statistical analyses

We used descriptive statistics of mean values and standard deviation to analyze the demographic information of individuals with SSA.

To determine the association between the level of social anxiety and structural neural correlates, we performed voxel-wise correlational analyses using FreeSurfer between the LSAS total scores and the GMV, CT, and SA of whole brain regions. We further analyzed the correlation between the LSAS performance anxiety scores and structural brain measures similarly. Age, sex, and ICV were used as covariates in the model; by controlling for these variables, we conducted a Monte Carlo simulation to correct for multiple comparisons using CWP and assessed the unique contributions of social anxiety levels (or performance anxiety levels) and brain regions to the outcome measures.

According to the results of the voxel-wise analysis, the mean GMV, CT, and SA values were extracted from regions with a significant correlation with the LSAS total scores or the LSAS performance anxiety scores. We performed exploratory Pearson’s correlation analyses to assess the linear relationships between the mean GMV, CT, and SA values in brain regions related to social anxiety level (or performance anxiety level) and other clinical features (e.g., GAD-7, BAI, and BDI-II) among individuals with SSA. The significance level of the *α* coefficient was set to 0.05 for statistical significance. Furthermore, we performed a Bonferroni correction in the exploratory correlation analyses (corrected *p*-value = 0.05/3 = 0.0167). We conducted additional statistical analyses using Statistical Package for the Social Sciences (SPSS) Windows version 27.0.

### Supplementary Information

Supplementary Information.

## Data Availability

All data used or analyzed during this study were included in this article. And further requests can be directed with a detailed description to the corresponding authors.
