# Supplementary material for: Altered cortical thickness of the superior frontal gyrus and fusiform gyrus in individuals with subthreshold social anxiety
Source: Sci Rep. 2023 Dec 9;13:21822. doi: 10.1038/s41598-023-49288-7 (PMC10710474; doi:10.1038/s41598-023-49288-7)
Supplement: Supplementary file 1 — Supplementary Information. [file 41598_2023_49288_MOESM1_ESM.docx]

**Supplementary Materials**

**Supplementary Table 1.** Pearson’s correlation analyses between cortical thickness in peak clusters related to social anxiety (or performance anxiety) and other clinical variables in individuals with SSA

|  | CT in the social anxiety-related peak cluster |  | CT in the performance anxiety-related peak clusters | |
| --- | --- | --- | --- | --- |
|  | SFG^a^ |  | SFG^b^ | FG^c^ |
| GAD-7 | *r*=0.317, *p*=0.010 |  | *r*=0.288, *p*=0.020 | *r*=-0.255, *p*=0.041 |
| BAI | *r*=0.150, *p*=0.235 |  | *r*=0.134, *p*=0.292 | *r*=-0.451, *p*<0.001 |
| BDI-II | *r*=0.155, *p*=0.222 |  | *r*=0.147, *p*=0.247 | *r*=-0.161, *p*=0.205 |

*Note:* MNI coordinates (*X*, *Y*, *Z*) [SFG^a^ (-23.8, 16.5, 39.1), SFG^b^ (-23.4, 17.8, 38.3), FG^c^ (34.4, -48.9, -11.4)]. *Abbreviations*: SSA, subthreshold social anxiety; CT, cortical thickness; SFG, superior frontal gyrus; FG, fusiform gyrus; GAD-7, Generalized Anxiety Disorder-7; BAI, Beck Anxiety Inventory; BDI-II, Beck Depression Inventory-II.
